# Spanish Adaptation and Validation of the General Attitudes Towards Artificial Intelligence Scale (GAAIS)

**DOI:** 10.3390/ejihpe15110230

**Published:** 2025-11-11

**Authors:** Zeinab Arees, Sergio Guntín, Francisca Fariña, Mercedes Novo

**Affiliations:** 1 Forensic Psychology Service, Faculty of Psychology, University of Santiago de Compostela, 15782 Santiago de Compostela, Spain; zeinab.arees@rai.usc.es (Z.A.); sergioguntin.mareque@usc.es (S.G.); 2 UNESCO Chair on Transformative Education: Science, Communication and Society, University of Vigo, 36005 Pontevedra, Spain; francisca@uvigo.es

**Keywords:** instrument adaptation, psychometric properties, reliability, validity, Confirmatory Factor Analysis (CFA), Spanish population, public attitudes, social perception, trust, technology

## Abstract

Artificial intelligence (AI) is generating a profound and quick transformation in several areas of knowledge, as well as in industry and society on a global scale, and is considered one of the most significant technological advances of the present era. Understanding citizens’ attitudes toward AI is essential forguiding its development and implementation. To achieve this, valid and reliable instruments are needed to assess attitudesin different sociocultural contexts. With this objective, the General Attitudes towards Artificial Intelligence Scale (GAAIS) was adapted to Spanish. The sample comprised 644 participants: 327 men and 316 women, aged between 18 and 78 years (*M* = 33.06, *SD* = 14.91). The original two-factor structure (Positive GAAIS and Negative GAAIS) was validated using Confirmatory Factor Analysis (CFA). Both the fit indices and the internal consistency of the scale were adequate. Furthermore, the validity of the measure (i.e., convergent and discriminant) and the invariance of the model were confirmed. The analyses performed support the adequacy of the model and, therefore, the usefulness of the instrument, considering the ambivalence that people often experience regarding AI. The limitations of the study and the implications for the design of public policies and intervention strategies that promote the ethical, equitable, and socially responsible use of AI are discussed in this study.

## 1. Introduction

Artificial intelligence (AI) can be considered one of the most important technological advances of the 21st century (Brynjolfsson & McAfee, 2014). Despite the lack of a universally agreed-upon definition of artificial intelligence (Wang, 2019), AI can be described as “systems that display intelligent behavior by analyzing their environment and taking actions—with some degree of autonomy—to achieve specific goals” (p. 1) (High-Level Expert Group on Artificial Intelligence of the European Commission, 2019), a proposal with sufficient scope and capacity to adapt to future changes (Sheikh et al., 2023). In contrast tonatural intelligence, AI controls the effects of cognitive and emotional biases on judgment and decision-making (Fariña et al., 2002; Selaya et al., 2024).

AI has spread widely, causing a deep transformation in different fields, including industry, healthcare, energy, environment, education, finance, transportation, judicial settings, and logistics (Brynjolfsson & McAfee, 2014; Chui et al., 2018; Leal et al., 2023). For example, in the healthcare field, AI diagnoses diseases more accurately and prescribes personalized treatments, improving the quality of healthcare (Haenlein & Kaplan, 2021; Johnson et al., 2021). In the business sector, it optimizes strategic business decision-making by analyzing immense amounts of data and extracting precise patterns, improving planning and management (Dwivedi et al., 2021). It is also effective in education, where it developspersonalized learning systems that takeinto account students’ needs, thereby reducing educational gaps and improving teaching quality (Zhai et al., 2022). In the legal field, it can contribute to improving judicial processes and decision-making, and in industry, it facilitates the intelligent automation of processes, especially in the field of mechanics (Valavanidis, 2023). There have even been widespread advances in revolutionary AI applications related to people’s daily lives, such as personal assistants (e.g., Alexa, Siri; Brill et al., 2019), self-driving cars (Hong et al., 2021; Manoharan, 2019), or social robots (Glas et al., 2016; Hentout et al., 2019).

### 1.1. Attitudes Towards AI

Despite being hailed as one of the world’s most important innovations (Valavanidis, 2023), artificial intelligence (AI) often elicits ambivalent public attitudes, particularly regarding issues like privacy, unemployment, and social justice (Zhu et al., 2025). In Spain, for example, the 2024 National Survey on the Social Perception of Science and Technology (Fundación Española para la Ciencia y la Tecnología, 2025) revealed widespread enthusiasm for AI. The survey indicated high usage rates (over 80%) and a clear recognition of AI’s benefits, with improved process efficiency being the most cited advantage (49.8%). However, this enthusiasm is coupled with significant public distrust. Many citizens admit to not understanding how AI works and express concerns about personal data security (42.4%), a false sense of security in AI systems (32.6%), and increased control by governments (28.9%) and companies (16.7%).

These concerns are echoed across various sectors. In the public sphere, AI raises fears of increased government surveillance and reduced personal freedoms (Sobrino-García, 2021). In the workplace, it may drive structural changes that create new opportunities while threatening traditional jobs (Méndez et al., 2008). In education, AI can enhance personalized learning but may also diminish human interaction (Galindo-Domínguez et al., 2023). Similarly, in the justice system, it could speed up legal decision-making but also risks undermining human legal analysis and the integrity of judicial outcomes (Lozano et al., 2021). Furthermore, AI is not immune to perpetuating gender biases, making it essential to identify and address these disparities (Grassini & Ree, 2023; Arees et al., 2024).

Public attitudes toward AI will not only affect its immediate acceptance and implementation but will also play a crucial role in shaping its future development (Zhu et al., 2025). Understanding these attitudes helps identify fears and uncertainties, enabling organizations to address these concerns and foster greater technology adoption (Kim & Lee, 2024; World Economic Forum, 2024). Moreover, studying public attitudes is key to guiding AI’s development responsibly and informing public training and digital literacy initiatives in response to AI’s rapid advancement (Montag & Ali, 2024). Ultimately, this process reveals critical ethical concerns, which areessential for ensuring that AI development remains aligned with human values (Mittelstadt, 2019; United Nations System Chief Executives Board for Coordination, 2022; Uzougbo et al., 2024).

### 1.2. AI Attitude Measurement

Models of acceptance and adoption of new technologies have been fundamental to understanding how individuals interact with emerging tools in different contexts, including artificial intelligence (e.g., Ali et al., 2024). Among the most influential are the Technology Acceptance Model (TAM; Davis, 1989; Davis et al., 1989), which emphasizes perceived usefulness and perceived ease of use, and the Unified Theory of Acceptance and Use of Technology (UTAUT; Venkatesh et al., 2003), which extends TAM by incorporating factors such as social influence and facilitating conditions.

These frameworks make it possible to identify the key determinants of the intention to use and attitude toward technologies, serving as the foundation for developing specific instruments that measure attitudes and perceptions of AI, as well as for interpreting how psychological and contextual variables influence its acceptance and effective use.

Several instruments have been developed to assess public attitudes toward AI. Among the most widely cited is the General Attitudes towards Artificial Intelligence Scale (GAAIS; Schepman & Rodway, 2020), which has been validated across diverse cultural contexts (Şahin & Yıldırım, 2024). While shorter scales have emerged recently—such as the AI Attitude Scale (AIAS-4; Grassini, 2023), Attitudes Towards AI (ATAI; Sindermann et al., 2021), and the Threats of Artificial Intelligence (TAI) scale (Kieslich et al., 2021)—these often focus on specific dimensions like fear. In contrast, the GAAIS is well-suited for capturing the full complexity of individual attitudes, addressing the limitations of shorter scales that may not fully represent these views or may overemphasize negative perceptions (Zhu et al., 2025).

In their initial validation study, Schepman and Rodway (2020) used Exploratory Factor Analysis (EFA) to identify two core dimensions of attitudes toward AI: a positive dimension reflecting its social and personal utility, and a negative dimension capturing common concerns. The scale demonstrated strong psychometric properties, including good convergent and discriminant validity against existing measures. The authors also found that comfort with technology was a strong predictor of favorable AI attitudes, while perceived capability was a weaker predictor. Additionally, they noted that people viewed AI more positively for data-intensive applications (e.g., astronomy, cybersecurity) but more negatively in domains requiring human judgment (e.g., psychological counseling, arts) (Schepman & Rodway, 2020).

In a subsequent study, the same authors performed a Confirmatory Factor Analysis (CFA), re-confirming the two-factor structure of the GAAIS (Schepman & Rodway, 2023). They also explored its relationship with the Big Five personality traits, finding that higher levels of introversion, conscientiousness, agreeableness, and general trust were all associated with more positive or tolerant attitudes toward AI.

The GAAIS has since been translated into multiple languages and validated in countries such as Turkey (Kaya et al., 2022), Korea (Seo & Ahn, 2022), Italy (Sacco et al., 2025), and China (Zhu et al., 2025). A shorter version has also been validated across several European nations (Bergdahl et al., 2023).

### 1.3. The Present Study

Despite the exponential growth of artificial intelligence and the cultural factors that shape its social perception, there are no instruments in the Spanish context capable of measuring public attitudes toward AI while accounting for their ambivalent nature. Therefore, this study aims to adapt and validate the Spanish version of the GAAIS, analyzing its psychometric properties to offer a reliable tool that supports empirical research and future investigations.

## 2. Materials and Methods

### 2.1. Participants

The selection of participants was carried out using a convenience approach, and the sample was expanded using the snowball method. The questionnaire was administered in digital format via an online web form. The invitation to participate was distributed through social networks, and participants were also asked to share the link with others, following the logic of chain sampling. Data were collected between February and December 2024. Participation was voluntary, and no compensation was provided. After listwise deletion of cases with missing values, the final sample consisted of 644 individuals. The sample was 50.8% male (*n* = 327) and 49.1% female (*n* = 316), with one participant (0.2%) selecting “Other.” Ages ranged from 18 to 78 years (*M* = 33.06, *SD* = 14.91). Regarding educational attainment, 5.6% (*n* = 36) had completed primary education, 29.2% (*n* = 188) had completed secondary education, and 65.1% (*n* = 419) had a university-level education.

Furthermore, participants were asked whether they worked or studied in a field related to artificial intelligence, new technologies, computing, or big data. The results showed that 7.3% of individuals (*n* = 47) had a direct link to the field of artificial intelligence, 16.6% (*n* = 107) indicated that their current occupation was related to new technologies, 21.1% (*n* = 136) indicated a link to the field of computing, and 6.7% (*n* = 43) stated that their work activity was related to big data.

### 2.2. Procedure and Data Analysis

The 20 items of the General Attitudes Towards Artificial Intelligence Scale (GAAIS; Schepman & Rodway, 2023) were translated from English to Spanish. A committee of experts then reviewed the translated items to ensure semantic and cultural equivalence, followed by a back-translation to verify fidelity to the original scale.

All statistical analyses were performed in R (RStudio IDE, Version 2024.12.1). First, an exploratory data analysis was performed on all study variables. This included calculating basic descriptive statistics (see Appendix B) and applying relevant normality tests to examine the distributions and identify potential violations of assumptions. Items on the GAAIS and the Attitude towards Artificial Intelligence (ATAI) scale—selected to assess convergent and discriminant validity—were reverse-coded where necessary. Subsequently, Spearman’s rho (ρ) correlations were calculated for all item pairings between the two scales.

To validate the two-factor structure proposed by Schepman and Rodway (2023) through Confirmatory Factor Analysis (CFA), different structural equation models were tested using the lavaan package (Rosseel, 2012). This was carried out based on a polychoric correlation matrix (Bandalos & Finney, 2018), using the Weighted Least Squares Mean and Variance Adjusted (WLSMV) estimator, as it is a robust estimator, especially suitable for ordinal data that does not necessarily fit a multivariate normal distribution. Model fit was assessed using the chi-square and degree of freedom ratio (χ^2^/df), the comparative fit indices Comparative Fit Index (CFI; Bentler, 1990) and the Tucker–Lewis Index (TLI; Tucker & Lewis, 1973), and the absolute fit indices, taking into account the root mean square error of approximation (RMSEA; Steiger, 1990) and the standardized root mean square residual (SRMR; Bentler, 1995). For the interpretation of results, values equal to or less than three were considered adequate for the chi-square ratio per degree of freedom (χ^2^/df ≤ 3). Regarding the comparative fit indices (CFI and TLI), values greater than 0.90 were considered indicative of a good fit (Hu & Bentler, 1999). For the absolute fit indices, RMSEA values equal to or less than 0.05 were used as a reference to describe a good model fit, and values between 0.05 and 0.08 were used to indicate an adequate fit (Browne & Cudeck, 1993). For the SRMR, a threshold of 0.08 was established to distinguish models characterized by a good fit to the data from models with a poorer fit, as this index is sensitive to model misspecification (Hu & Bentler, 1999).

Scale reliability was evaluated using Cronbach’s α (Cronbach, 1951), ordinal α (Zumbo et al., 2007), and McDonald’s ω (McDonald, 1999) coefficients. Convergent and discriminant validity were assessed by calculating the Average Variance Extracted (AVE), applying the Fornell–Larcker criterion (1981), and computing the Heterotrait–Monotrait Ratio (HTMT; Henseler et al., 2015). Additionally, Spearman’s rho correlations between the GAAIS and ATAI dimensions were analyzed using a permutation test to determine if correlations between theoretically related dimensions were significantly stronger than those between less related dimensions.

Finally, a cross-validation procedure was implemented to assess the stability of the factor solution across different subsamples. A factorial invariance analysis was also performed between groups by sex to examine whether the factor structure remained constant across the four levels of invariance: configural, metric, scalar, and strict. Models were estimated using multigroup confirmatory factor analysis with the WLSMV estimator and theta parameterization, which allows explicit estimation of residual variances required for testing strict invariance. When comparing the different invariance models, changes in the fit indices were taken as evaluation criteria. Specifically, differences in CFI (ΔCFI ≤ 0.01), RMSEA (ΔRMSEA ≤ 0.015), and SRMR (ΔSRMR ≤ 0.01 for metric invariance and ΔSRMR ≤ 0.03 for scalar invariance) were considered, following the recommendations of Chen (2007).

### 2.3. Measures

The GAAIS contains 20 items divided into two dimensions: Positive GAAIS (12 items) and Negative GAAIS (8 items). The first dimension reflects perceived opportunities, benefits, and positive emotions related to AI, while the negative dimension focuses on concerns and negative emotions. Additionally, the authors include an attention control element to verify whether the questions had been read, to exclude participants who may have responded randomly. Each item is rated on a five-point Likert scale ranging from 1 (“strongly disagree”) to 5 (“strongly agree”). Items belonging to the Negative GAAIS are reverse-scored so that, in both subscales, higher scores represent more positive attitudes toward artificial intelligence. The mean score of each subscale can be used as an indicator of the respective attitudinal dimension, and the authors do not recommend computing a single total score for the entire scale. Thus, higher values in the Positive GAAIS indicate greater enthusiasm and perceived utility of AI (e.g., “Artificial intelligence can provide new economic opportunities for this country”), whereas lower scores in the Negative GAAIS reflect stronger concerns and distrust toward it (e.g., “Artificial intelligence is used to spy on people”).

Similarly, the Attitude towards Artificial Intelligence (ATAI) scale (Sindermann et al., 2021) was used to assess convergent validity. It consists of five items across two dimensions: Acceptance and Fear. For both the GAAIS and ATAI, responses were collected on a five-point Likert-type scale ranging from 1 (“Strongly Disagree”) to 5 (“Strongly Agree”).

## 3. Results

### 3.1. Descriptive Results

Spearman’s rho correlation matrix among the items showed values ranging from −0.119 to 0.598, with several non-significant associations. These results suggest considerable heterogeneity among some items, but do not indicate significant multicollinearity. Items 3 and 5 had the highest number of non-significant correlations. Items within the Positive GAAIS (PG) dimension tend to be more closely related to each other, with higher correlations within the dimension. This pattern also held true for the Negative GAAIS (NG) dimension. In contrast, the correlations obtained between items from the Positive GAAIS (PG) and the Negative GAAIS (NG) dimensions were notably lower.

### 3.2. Validity

A two-factor correlated model based on the original scale structure was evaluated, which showed unsatisfactory fit indices: χ^2^ = 848.234, df = 169, *p* < 0.001, CFI = 0.851, TLI = 0.832, RMSEA = 0.082, and SRMR = 0.069. Based on the analysis of the item factor loadings and modification indices, an adjusted model was proposed. In this second version, four items (i3, i5, i6, and i13) were eliminated due to their low factor loadings (λ < 0.40) and their limited contribution to the construct. Similarly, the high residual variances associated with these items were a factor in their removal, reflecting a significant degree of error not explained by the model and compromising its psychometric adequacy. Furthermore, acorrelation between the errors of item pairs i4-i11, i7-i18, and i9-i10 was allowed due to their conceptual similarities.

In this second scenario, the results of the chi-square test (χ^2^ = 388.180, df = 100, *p* < 0.001) indicated a better model fit compared to the initial model and the base model, which assumes independence between variables. Although the result was significant (*p* < 0.001), it is important to note that this statistic is especially sensitive to sample size and can exaggerate significance in large samples, as is the case here, so it should be interpreted with caution. However, the lower value obtained in the factorial model compared to the base model suggests a considerably better fit. The χ^2^/dfratio (388.180/100) was 3.88, slightly above the conventional cutoff for a good fit (χ^2^/df ≤ 3), but within the range often regarded as acceptable when other fit indices are satisfactory. Regarding the absolute fit indices (see Table 1), the Root Mean Square Error of Approximation (RMSEA) showed a value of 0.069, with a 95% confidence interval between 0.059 and 0.078, and the standardized root mean square residual (SRMR) was 0.051. The RMSEA value can be considered adequate, while the SRMR indicates a good model fit. Similarly, regarding the comparative fit indices, the CFI obtained a value of 0.926, and the TLI a value of 0.911. Both values confirm the good fit of the model. As part of the analysis, a unifactorial model was also specified to assess its fit compared to the previous model. As shown in Table 1, this model had the poorest fit of the three (χ^2^ = 2250.817, df = 170, *p* < 0.001, CFI = 0.630, TLI = 0.586, RMSEA = 0.138, SRMR = 0.117).

**Figure 1:**
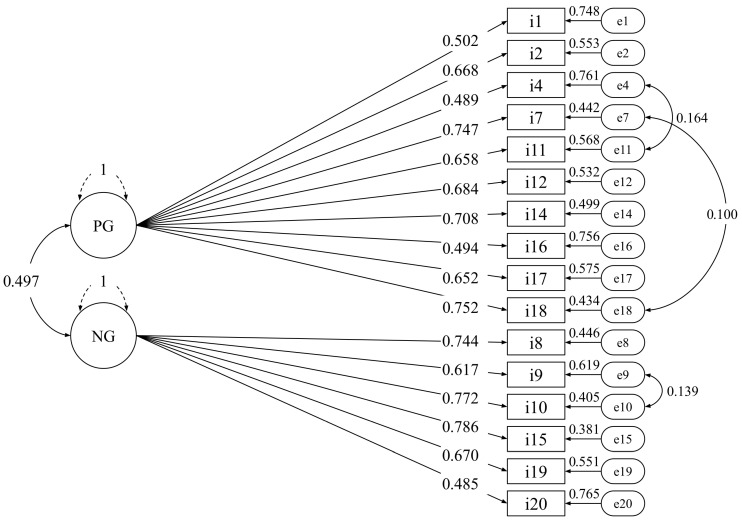
Factor diagram. Note. PG (Positive GAAIS) and NG (Negative GAAIS) refer to latent variables, whereas the items that make up the factors are indicators of these variables. The number of each item corresponds to the order in which they were presented to the participants.

The final model (see Figure 1) consisted of 16 items, 10 for the Positive GAAIS subscale and 6 for the Negative GAAIS subscale. All factor loadings were statistically significant, with values ranging from 0.485 to 0.786 (all λs > 0.4).

### 3.3. Reliability

Regarding the scale’s reliability, Cronbach’s α was 0.854, supporting an adequate internal consistency. As for the subscales, Cronbach’s α was 0.840 for the Positive GAAIS and 0.808 for the Negative GAAIS. The ordinal alpha coefficient was 0.879 for the total scale, 0.873 for the positive subscale, and 0.838 for the negative subscale. Finally, the McDonald’s ω was 0.873 for the overall scale, 0.836 for the Positive GAAIS, and 0.804 for the Negative GAAIS. Overall, these indices indicate that both the scale and the subscales exhibit adequate and consistent reliability, supporting their use for assessing the construct (see Table 2).

### 3.4. Convergent and Discriminant Validity

The model showed limited internal convergent validity (see Table 2). The AVE values were slightly below the recommended threshold of 0.50 (Positive GAAIS = 0.413; Negative GAAIS = 0.472; Total GAAIS = 0.435). The average item coefficient of determination (R^2^) was consistent with these results (0.42, 0.49, and 0.45, respectively), indicating that the latent factors explained 42% and 49% of the variance in their corresponding items.

In addition, internal discriminant validity was adequate. According to the Fornell and Larcker (1981) criterion, the square root of the AVE for each construct was greater than the correlations between constructs (Positive GAAIS = 0.643 > 0.480; Negative GAAIS = 0.687 > 0.480). The HTMT index (Henseler et al., 2015) was 0.472, below the critical threshold of 0.85, indicating adequate discrimination between the two factors.

Furthermore, Spearman’s rho correlation (ρ) was calculated between the GAAIS subscales (Positive and Negative) and the dimensions of the Attitude Towards Artificial Intelligence (ATAI; Sindermann et al., 2021), Acceptance, and Fear. Overall, there was a strong correlation between the two instruments (ρ = 0.766, *p* < 0.001). This was also true for the theoretically related sub-dimensions. Thus, Positive GAAIS and Acceptance (ATAI) showed a correlation of 0.657 (*p* < 0.001), and Negative GAAIS and Fear (ATAI) had a correlation of 0.800 (*p* < 0.001). Regarding discriminant validity, the lowest correlations in the matrix correspond to the relationship between the Positive GAAIS subscale and Fear (ATAI), as well as between the Negative GAAIS subscale and Acceptance (ATAI). In the first case, a value of ρ = 0.368, *p* < 0.001, was obtained, and in the second, a correlation of ρ = 0.460 (*p* < 0.001) was obtained. Furthermore, statistically significant differences were observed (see Table 3) between the correlations of the theoretically related dimensions (PG ↔ Acceptance, NG ↔ Fear) and the correlations of the less related dimensions (PG ↔ Fear, NG ↔ Acceptance).

### 3.5. Evaluation of Factorial Solution Stability

To assess the consistency of the scale’s factor structure, the total sample (*N* = 644) was randomly divided into two subsamples (using an odd-even split for cross-validation; *n*_1_ = 322, *n*_2_ = 322), and a Confirmatory Factor Analysis (CFA) was performed on each half. The structure identified above was replicated in both subsamples, yielding fit indices that were consistent with the full sample and the subsamples, albeit with slight variations (see Table 4). With the reduced sample size, the χ^2^/dfratio values decreased considerably compared to the initial analysis. The absolute and comparative fit indices remained within acceptable ranges in all cases. Although there were slight variations, the results do not seriously compromise the model’s stability. Factor loadings were similar between the two analyses, with standardized loadings ranging from 0.38 to 0.80. These results suggest that the scale’s factor structure is consistent and generalizable to different subsamples, reinforcing its structural validity.

Finally, the model’s invariance across sexes was tested. Adequate fit indices were obtained for the initial measurement models for men (χ^2^ = 295.215, df = 100, *p* < 0.001, CFI = 0.900, RMSEA = 0.077, SRMR = 0.071) and women (χ^2^ = 227.463, df = 100, *p* < 0.001, CFI = 0.927, RMSEA = 0.072, SRMR = 0.056), prior to testing measurement invariance. As shown in Table 5, the results support full factorial invariance. The fit indices are adequate at all levels of analysis, and the differences observed between them do not exceed the cutoff points typically used to compare the different types of invariance (Chen, 2007). Thus, the instrument measures the construct equally for men and women in terms of factor structure, loadings, intercepts, and error variances, allowing for valid comparisons between the two groups.

## 4. Discussion

It is essential to have valid and reliable instruments to assess public attitudes across different sociocultural contexts. To this end, this study adapted the General Attitudes towards Artificial Intelligence Scale (GAAIS; Schepman & Rodway, 2020) into Spanish (see Appendix A). The results from the different analyses supported the original two-factor structure (Positive GAAIS and Negative GAAIS). The final model had acceptable fit indices and adequate internal consistency. To obtain this level of fit, four items were eliminated from the original scale due to their poor psychometric performance and limited theoretical contribution. From a conceptual standpoint, the removed items addressed aspects closely related to those already represented in the retained items, suggesting that their exclusion contributes to a more streamlined model without compromising construct coverage. The final scale proposed for use in Spanish consists of 16 items divided into two subscales: Positive GAAIS (10 items) and Negative GAAIS (6 items).

The results showed limited convergent validity (AVE < 0.50). However, this could be due to the nature of the construct being measured, since attitudes involve cognitive, affective, and behavioral elements (Rosenberg & Hovland, 1960) that are not necessarily perfectly aligned but are part of the same general construct. Thus, different facets of one’s attitude toward AI can be relatively independent or even conflicting. A good example would be individuals who believe that AI is useful but who nevertheless fear it and do not want to use it in their daily lives. Furthermore, the items that make up the scale cover a wide range of ideas applied to different contexts (social benefits, work efficiency, personal comfort with using AI), an aspect that could also contribute to the scale’s low internal convergent validity. In contrast, adequate discriminant validity was found, supporting the differentiation between both dimensions (Positive GAAIS and Negative GAAIS). Moreover, the correlations between the GAAIS and ATAI subscales showed statistically significant differences between theoretically related and less related dimensions. Overall, these results support the instrument’s validity in distinguishing between the different positive and negative aspects of artificial intelligence. The factor solution was found to be consistent across different subsamples, and full factorial invariance was confirmed across gender, indicating that the scale measures the construct equally in men and women, allowing for valid comparisons between groups without measurement bias.

The moderate correlation between the two factors is consistent with that reported in other adaptations (Sacco et al., 2025; Schepman & Rodway, 2020, 2023; Zhu et al., 2025). Regarding reliability, the coefficients obtained are in line with those reported by Şahin and Yıldırım (2024) in their meta-analytic review, which integrated data from 19 independent studies conducted across different regions of Asia, Europe, and the Americas. The authors reported Cronbach’s alpha values of 0.881, 0.828, and 0.863 for the total, negative, and positive subscales, respectively, figures that are like those obtained in the present study (see Table 2).

In comparison with other instruments designed to assess attitudes toward artificial intelligence, such as the ATAI (Sindermann et al., 2021), the bifactorial structure of the GAAIS corresponds to the dimensions of acceptance and fear proposed in that questionnaire. Thus, both instruments consistently capture the coexistence of positive and negative components in attitudes toward AI. However, the GAAIS provides a broader and more balanced assessment by including both cognitive beliefs and emotional responses without overemphasizing the negative affective component, as occurs in the ATAI, where three of the five items focus on fear. Meanwhile, the AIAS (Grassini, 2023) seeks a brief and unidimensional measure of general attitude toward artificial intelligence, which facilitates its use in large samples but limits its ability to capture the attitudinal ambivalence that characterizes this construct. Overall, the Spanish version of the GAAIS preserves the theoretical complexity and sensitivity of the original scale, offering a balance between conceptual breadth and psychometric robustness that distinguishes it from other available instruments.

Regarding the scoring and interpretation of the scale, it is recommended to follow the original instructions provided by the authors (Schepman & Rodway, 2020). Items belonging to the Negative GAAIS are reverse-scored so that higher values in both subscales represent more positive attitudes toward artificial intelligence. The mean scores of each subscale are used as indicators of positive and negative attitudes, where higher scores on the Positive GAAIS reflect greater enthusiasm and perceived utility, whereas lower scores on the Negative GAAIS indicate stronger concerns and distrust.

### 4.1. Implications

The rapid incorporation of artificial intelligence into various areas of social and professional life poses new challenges for psychological research. In this context, having valid and reliable instruments is essential to understand how people perceive, evaluate, and accept these technologies, as well as to guide their responsible and equitable implementation.

Having psychometrically sound instruments is crucial not only to better understand social perceptions surrounding artificial intelligence but also to inform the design of public policies and intervention strategies that promote the ethical, equitable, and socially responsible use of these emerging technologies (Lozano et al., 2021; Stein et al., 2024; Valavanidis, 2023; World Economic Forum, 2024). From a practical perspective, the validation of the GAAIS provides a reliable tool to assess attitudes toward AI, with potential for application and comparison across different professional contexts in future research. One of its most relevant contributions is its ability to capture the ambivalence that characterizes many of these attitudes, combining positive perceptions related to AI’s innovative potential with ethical, labor, and social concerns. This feature enables a more nuanced understanding of the factors influencing the acceptance or rejection of these technologies and can inform the design of training programs, institutional strategies, and awareness campaigns aimed at fostering informed and responsible adoption. Likewise, the results obtained with the GAAIS may contribute to generating empirical evidence relevanttoinforming future public policies and regulatory frameworks that support the ethical and safe use of AI, as well as the protection of individuals’ fundamental rights (Sobrino-García, 2021).

From a theoretical perspective, this validation contributes to the development of the field of attitudes toward artificial intelligence by providing empirical evidence of its multidimensional structure and the coexistence of positive and negative evaluations toward the same technology. Furthermore, it opens the possibility of conducting cross-cultural comparisons and examining how variables such as education or technological experience shape the perception and acceptance of AI. Overall, the GAAIS contributes to strengthening a more comprehensive conceptual framework that helps to understand the conditions under which artificial intelligence can be integrated in a sustainable, ethical, and socially beneficial manner.

### 4.2. Limitations and Future Directions

It is important to consider several limitations that may affect the interpretation and generalization of the results. First, no prior pilot test was conducted, which could have helped identify potential difficulties in item comprehension, administration issues, or technical aspects that might affect data quality. Additionally, the sample used for this study is not necessarily representative of the entire population of Spain, since it was obtained through convenience sampling, which implies that the results should be interpreted with caution. Future studies should consider using probabilistic or stratified sampling methods to enhance external validity and representativeness. Likewise, the temporal stability of the instrument was not assessed through a test–retest procedure; this lack of evidence limits the conclusions regarding the instrument’s consistency over time.

Finally, another potential limitation is the method effect associated with reverse-worded items; that is, items worded negatively or whose direction is contrary to the general construct. This effect, which we could not control for in this study, can interfere with the instrument’s structure. Although these items are usually recoded to facilitate interpretation, they can add unwanted variance related to cognitive load, item comprehension, or systematic response patterns that reflect item format rather than construct content (Dueber et al., 2021; Tomás et al., 2012, 2013; Weijters et al., 2013). In this case, since the two dimensions are composed entirely of positively and negatively worded items, an artificial factor could have been generated by shared variance among the negative items (the Negative GAAIS subscale), which is not due to the construct itself. Therefore, future research should determine the extent to which this effect exists in the GAAIS and, if necessary, propose alternative models that are capable of controlling for it.

## 5. Conclusions

The Spanish adaptation of the GAAIS represents a significant advance in the assessment of attitudes toward artificial intelligence. The results support the original two-factor structure and, therefore, underscore the usefulness of the instrument in addressing the ambivalence that people often experience toward AI. The scale provides a solid tool for analyzing the social and emotional perceptions elicited by this emerging technology, and its use may help identify profiles of acceptance and resistance that can guide educational interventions or the development of more effective communication strategies regarding the use of AI.

## Figures and Tables

**Table 1:** Model fit indices.

Model	χ^2^/df	Absolute Fit Indices		Comparative Fit Indices	
		SRMR	RMSEA [95% CI]	CFI	TLI
Unifactorial	39.73	0.117	0.128 [0.121, 0.135]	0.630	0.586
Two factors	5.01	0.069	0.082 [0.075, 0.089]	0.851	0.832
Two factors (adjusted)	3.88	0.051	0.069 [0.059, 0.078]	0.926	0.911

Note. χ^2^/df = Chi-square divided by degrees of freedom (lower values indicate better model fit); SRMR = Standardized Root Mean Square Residual; RMSEA = Root Mean Square Error of Approximation; CFI = Comparative Fit Index; TLI = Tucker–Lewis Index. All reported fit indices are robust estimates, except for the SRMR, which is computed from the standardized residuals. See Appendix C for the corresponding non-robust (scaled) indices.

**Table 2:** Reliability of the scale and evidence of convergent and discriminant validity.

	Cronbach’s α	Ordinal α	McDonald’s ω	AVE	Fornell and Larcker’s Criterion		HTMT	Average R^2^
					PG	NG		
PG	0.840	0.873	0.836	0.413	0.643	0.480	0.472	0.42
NG	0.808	0.838	0.804	0.472	0.480	0.687	-	0.49
TG	0.854	0.879	0.873	0.435	-	-	-	0.45

Note. PG = Positive GAAIS; NG = Negative GAAIS; TG = Total GAAIS; AVE = Average Variance Extracted; HTMT = Heterotrait–Monotrait Ratio of Correlations; R^2^ = Average coefficient of determination of the items.

**Table 3:** Comparison of the observed correlations between the GAAIS and ATAI subscales.

Comparison	*ρ*_1_	*ρ*_2_	Δ*ρ*
(PG ↔ Acceptance) vs. (PG ↔ Fear)	0.657 ***	0.368 ***	0.289 ***
(PG ↔ Acceptance) vs. (NG ↔ Acceptance)	0.657 ***	0.460 ***	0.197 ***
(NG ↔ Fear) vs. (NG ↔ Acceptance)	0.800 ***	0.460 ***	0.341 ***
(NG ↔ Fear) vs. (PG ↔ Fear)	0.800 ***	0.368 ***	0.433 ***

Note. PG = Positive GAAIS; NG = Negative GAAIS; *ρ*_1_ = Spearman’s rho for the first set of subescales; *ρ*_2_ = Spearman’s rho for the second set of subescales; Δ*ρ* = Difference between both correlations; *** *p* < 0.001.

**Table 4:** Fit indices of the factor solution for *N*, *n*_1_, and *n*_2_.

Model	χ^2^/df	Absolute Fit Indices		Comparative Fit Indices	
		SRMR	RMSEA [95% CI]	CFI	TLI
*N*	3.88	0.051	0.069 [0.059, 0.078]	0.926	0.911
*n*_1_	2.77	0.064	0.071 [0.057, 0.085]	0.921	0.906
*n*_2_	2.19	0.055	0.074 [0.059, 0.088]	0.916	0.899

Note. χ^2^/df = Chi-square divided by degrees of freedom (lower values indicate better model fit); SRMR = Standardized Root Mean Square Residual; RMSEA = Root Mean Square Error of Approximation; CFI = Comparative Fit Index; TLI = Tucker–Lewis Index. All reported fit indices are robust estimates, except for the SRMR, which is computed from the standardized residuals. See Appendix C for the corresponding non-robust (scaled) indices.

**Table 5:** Fit indices of the factorial invariance models of the GAAIS across gender.

Model	χ^2^	df	CFI	RMSEA [95% CI]	SRMR	ΔCFI	ΔRMSEA	ΔSRMR
Men	295.215	100	0.900	0.077 [0.066, 0.089]	0.071	-	-	-
Women	227.463	100	0.927	0.072 [0.060, 0.084]	0.056	-	-	-
Configural	525.149	200	0.947	0.071 [0.064, 0.079]	0.063	-	-	-
Metric	516.917	214	0.951	0.066 [0.059, 0.073]	0.068	0.004	−0.005	0.004
Scalar	600.173	260	0.944	0.064 [0.057, 0.071]	0.065	−0.007	−0.002	−0.003
Strict	605.335	276	0.946	0.061 [0.054, 0.068]	0.065	0.002	−0.003	0.003

Note. χ^2^/df = Chi-square divided by degrees of freedom (lower values indicate better model fit); SRMR = Standardized Root Mean Square Residual; RMSEA = Root Mean Square Error of Approximation; CFI = Comparative Fit Index; Δ = Difference between the fit indices. Robust fit indices were reported for single-group models, whereas non-robust (scaled) fit indices were used for the measurement invariance analyses, as robust indices are not available or comparable across nested models estimated with the WLSMV estimator. See Appendix C for the corresponding non-robust (scaled) indices.

## Data Availability

The data presented in this study are available upon reasonable request from the corresponding author.

## Appendix A

**Table A1:** Original items from the GAAIS and their Spanish-translated versions.

Subscale	Number	Item
Positive	i1	Para transacciones rutinarias, prefiero interactuar con un sistema artificialmente inteligente que con un humano [For routine transactions, I would rather interact with an artificially intelligent system than with a human]
Positive	i2	La Inteligencia artificial puede brindar nuevas oportunidades económicas para este país [Artificial intelligence can provide new economic opportunities for this country]
Negative	i3	Las organizaciones utilizan la inteligencia artificial de forma poco ética [Organisations use artificial intelligence unethically] *
Positive	i4	Los sistemas de inteligencia artificial pueden ayudar a las personas a sentirse más felices [Artificially intelligent systems can help people feel happier]
Positive	i5	Estoy impresionado por lo que puede hacer la inteligencia artificial [I am impressed by what artificial intelligence can do] *
Negative	i6	Creo que los sistemas con inteligencia artificial cometen muchos errores [I think artificially intelligent systems make many errors] *
Positive	i7	Me interesa utilizar sistemas de inteligencia artificial en mi vida diaria [I am interested in using artificially intelligent systems in my daily life]
Negative	i8	La inteligencia artificial me parece siniestra [I find artificial intelligence sinister]
Negative	i9	La inteligencia artificial podría tomar el control de las personas [Artificial intelligence might take control of people]
Negative	i10	Creo que la inteligencia artificial es peligrosa [I think artificial intelligence is dangerous]
Positive	i11	La inteligencia artificial puede tener impactos positivos en el bienestar de las personas [Artificial intelligence can have positive impacts on people’s wellbeing]
Positive	i12	La inteligencia artificial es apasionante [Artificial intelligence is exciting]
Attention Check	A	Le agradecería que seleccionara “Totalmente de acuerdo” [To show that you are reading this, please select Strongly agree]
Positive	i13	Un agente con inteligencia artificial sería mejor que un empleado en muchos trabajos rutinarios [An artificially intelligent agent would be better than an employee in many routine jobs] *
Positive	i14	Hay muchas aplicaciones beneficiosas de la inteligencia artificial [There are many beneficial applications of artificial intelligence]
Negative	i15	Tiemblo de incomodidad cuando pienso en los usos futuros de la inteligencia artificial [I shiver with discomfort when I think about future uses of Artificial Intelligence]
Positive	i16	Los sistemas artificialmente inteligentes pueden funcionar mejor que los humanos [Artificially intelligent systems can perform better than humans]
Positive	i17	Gran parte de la sociedad se beneficiará de un futuro lleno de inteligencia artificial [Much of society will benefit from a future full of Artificial Intelligence]
Positive	i18	Me gustaría utilizar la inteligencia artificial en mi propio trabajo [I would like to use Artificial Intelligence in my own job]
Negative	i19	La gente como yo sufrirá si se utiliza cada vez más la inteligencia artificial [People like me will suffer if artificial intelligence is used more and more]
Negative	i20	La inteligencia artificial se utiliza para espiar a las personas [Artificial intelligence is used to spy on people]

Note. * Items removed from the Spanish version of the scale.

## Appendix B

**Table A2:** Item response frequencies and descriptive statistics of the GAAIS and ATAI scale.

Item	Frequency					Mean	SD	Skewness	Kurtosis
	TD [1]	SD [2]	NA/ND [3]	SA [4]	TA [5]				
PG—i1	267	159	87	98	33	2.179	1.261	0.734	−0.702
PG—i2	38	52	110	272	172	3.758	1.111	−0.902	0.218
NG—i3	22	52	194	245	131	3.638	1.003	−0.527	−0.023
PG—i4	82	116	203	204	39	3.003	1.118	−0.294	−0.746
PG—i5	14	13	43	172	402	4.452	0.874	−1.981	4.218
NG—i6	14	106	195	257	72	3.415	0.963	−0.301	−0.475
PG—i7	106	85	150	232	71	3.120	1.257	−0.392	−0.938
NG—i8	82	121	140	194	107	3.191	1.276	−0.249	−1.025
NG—i9	124	112	116	194	98	3.047	1.362	−0.181	−1.236
NG—i10	49	76	106	233	180	3.651	1.217	−0.719	−0.437
PG—i11	25	26	105	352	136	3.851	0.930	−1.168	1.713
PG—i12	38	54	206	196	150	3.568	1.111	−0.493	−0.298
PG—i13	154	203	107	147	33	2.537	1.222	0.319	−1.065
PG—i14	15	22	63	326	218	4.102	0.879	−1.330	2.378
NG—i15	97	104	150	186	107	3.158	1.302	−0.253	−1.038
PG—i16	108	174	157	166	39	2.773	1.180	0.053	−1.016
PG—i17	64	107	150	232	91	3.278	1.189	−0.400	−0.759
PG—i18	129	101	176	161	77	2.932	1.298	−0.096	−1.082
NG—i19	92	132	207	135	78	2.961	1.213	0.001	−0.854
NG—i20	57	92	133	265	97	3.393	1.166	−0.567	−0.547
ATAI—i1	120	118	129	203	74	2.989	1.306	−0.173	−1.173
ATAI—i2	73	150	196	199	26	2.930	1.074	−0.216	−0.817
ATAI—i3	197	150	164	100	33	2.413	1.214	0.381	−0.905
ATAI—i4	28	49	140	337	90	3.640	0.961	−0.922	0.753
ATAI—i5	16	36	63	238	291	4.168	0.985	−1.320	1.426

Note. *N* = 644. GAAIS = General Attitudes towards Artificial Intelligence Scale; ATAI = Attitude Towards Artificial Intelligence scale; PG = Positive GAAIS; NG = Negative GAAIS; TD = Totally Disagree; SD = Somewhat Disagree; NA/ND = Neither Agree nor Disagree; SA = Somewhat Agree; TA = Totally Agree; SD = Standard Deviation. All values were calculated without recoding the reverse-worded items.

## Appendix C

**Table A3:** Non-robust (scaled) fit indices for all estimated models.

Model	RMSEA [95% CI]	CFI	TLI
Unifactorial	0.138 [0.132, 0.144]	0.717	0.684
Two factors	0.079 [0.073, 0.085]	0.908	0.896
Two factors (adjusted)	0.067 [0.059, 0.075]	0.954	0.945
Evaluation of factorial solution stability			
*n*_1_	0.074 [0.062, 0.087]	0.942	0.930
*n*_2_	0.061 [0.048, 0.074]	0.963	0.956
Evaluation of factorial invariance across gender			
Man	0.079 [0.068, 0.089]	0.917	0.900
Woman	0.063 [0.052, 0.073]	0.965	0.958

Note. RMSEA = Root Mean Square Error of Approximation; CFI = Comparative Fit Index; TLI = Tucker–Lewis Index.
